# Collective Variable for Metadynamics Derived From AlphaFold Output

**DOI:** 10.3389/fmolb.2022.878133

**Published:** 2022-06-13

**Authors:** Vojtěch Spiwok, Martin Kurečka, Aleš Křenek

**Affiliations:** ^1^ Department of Biochemistry and Microbiology, Faculty of Food and Biochemical Technology, University of Chemistry and Technology, Prague, Czechia; ^2^ Institute of Computer Science, Masaryk University, Brno, Czechia

**Keywords:** AlphaFold, protein folding, protein structure prediction, metadynamics, deep learning, free-energy simulation, collective variable

## Abstract

AlphaFold is a neural network–based tool for the prediction of 3D structures of proteins. In CASP14, a blind structure prediction challenge, it performed significantly better than other competitors, making it the best available structure prediction tool. One of the outputs of AlphaFold is the probability profile of residue–residue distances. This makes it possible to score any conformation of the studied protein to express its compliance with the AlphaFold model. Here, we show how this score can be used to drive protein folding simulation by metadynamics and parallel tempering metadynamics. Using parallel tempering metadynamics, we simulated the folding of a mini-protein Trp-cage and *β* hairpin and predicted their folding equilibria. We observe the potential of the AlphaFold-based collective variable in applications beyond structure prediction, such as in structure refinement or prediction of the outcome of a mutation.

## 1 Introduction

The introduction of the AlphaFold tool, and especially its second version (Jumper et al., 2021), represents a significant improvement in the prediction of 3D structures of proteins. In CASP14 structure prediction competition, it outperformed other competitors, both in overall and local accuracy of predicted structures. It is very likely that AlphaFold will change the field of experimental structural biology, and this field will in the future focus on the function of proteins rather than the 3D structure itself.

Nevertheless, there are still limitations of AlphaFold such as limited capacity to predict the outcome of a point mutation, to predict structures of complexes with small-molecule ligands, to model an induced fit, and other limitations. This provides an opportunity for biomolecular simulations and their hybrid applications together with AlphaFold data.

AlphaFold 1 (Senior et al., 2020) was introduced in CASP13 blind structure prediction competition in 2018. One of the key premises in structure prediction by AlphaFold 1 is that coevolving residues are likely to be located close to each other in a 3D structure of a protein Sanchez-Pulido and Ponting (2021). The input of AlphaFold 1 is an amino acid sequence of the modeled protein. Next, homologous sequences are found in a database of sequenced proteins and aligned to yield a multiple sequence alignment. This alignment is converted to various coevolution features. Distributions of distances between residues are predicted from these features using an artificial neural network. This neural network is trained on proteins with known 3D structures from the Protein Data Bank (PDB) (Bernstein et al., 1977) and multiple sequence alignments with their sequenced homologs. Finally, the resulting distance distribution is used to predict the 3D structure of the modeled protein.

AlphaFold 2 has significantly improved accuracy compared to AlphaFold 1 by the introduction of novel neural network architectures and the integration of separated parts into a more compact neural network pipeline. Coevolution is modeled rather implicitly in this version. Both versions work with inter-residue distance maps. That is, AlphaFold 2 produces a tensor with dimension *N*x*N*x*M*, where *N* is the number of residues and *M* is the number of distance bins. This tensor stores the probabilities (expressed as logits) of a given residue pair being found at a given distance. The tensor makes it possible to evaluate any conformation of the modeled protein to estimate how much it fits the AlphaFold prediction. The level of this fitness was used in this work to drive a simulation of the protein, explore various conformations, and predict their equilibrium probabilities. For this purpose, we used the metadynamics method (Laio and Parrinello, 2002) and its combination with parallel tempering (Bussi et al., 2006) in explicitly modeled water. The concept was tested on two artificial fast-folding mini-protein tryptophan cage (Trp-cage) and *β* hairpin.

## 2 Materials and Methods

### 2.1 AlphaFold

The structure of the Trp-cage mini-protein (the construct TC5b) was predicted by AlphaFold 2 (initial release) (Jumper et al., 2021). The structure of *β* hairpin (the 16-residue C-terminal fragment of the G-B1 protein sequence GEWTYDDATKTFTVTE) was predicted by AlphaFold 2 (version 2.1.1). Both models were in excellent agreement with the experimentally determined structures (PDB ID 1L2Y (Neidigh et al., 2002) and 2GB1 (Gronenborn et al., 1991)), even if all homologous structures were excluded from the experimentally determined set of structures used by the program (by the option––max_template_date=1969-12-31).

### 2.2 Molecular Dynamics Simulation

All simulations were performed by using Gromacs 2021 (Abraham et al., 2015) patched with Plumed 2.7.2 (Tribello et al., 2014) modified to introduce the AlphaFold collective variable. The source code of those extensions is publicly available, and it will be added to Plumed in the near future. A Docker image of Gromacs built with this Plumed extension is available as ljocha/GROMACS: 2021-3.3 at Dockerhub. The image supports both single- and double-precision, SSE2/AVX2_265/AVX_512 instruction sets and GPU acceleration. It can be converted to singularity for use in HPC computing centers in a straightforward way.

In Trp-cage simulations, the system contained the mini-protein, 11,112 or 1,602 TIP3P water molecules (Jorgensen et al., 1973) (for metadynamics and parallel tempering metadynamics, respectively) and one chloride anion to neutralize the charge. In *β* hairpin simulations, the system contained the mini-protein, 11,136 or 1,625 TIP3P water molecules (for metadynamics and parallel tempering metadynamics, respectively) and three sodium cations to neutralize the charge.

The mini-proteins were modeled using Amber99SB-ILDN force field (Lindorff-Larsen et al., 2010). The time step was set to 2 fs, and all bonds involving hydrogen atoms were constrained by the LINCS algorithm (Hess et al., 1997). Electrostatic interactions were evaluated using the particle mesh Ewald method (Darden et al., 1993). Parrinello–Bussi thermostat (Bussi et al., 2007) and Parrinello–Rahman barostat (Parrinello and Rahman, 1981) were used to maintain constant temperature and pressure, respectively.

For metadynamics, the system was first optimized by the steepest descent method followed by 100-ps simulation in the NPT ensemble (constant number of particles, pressure, and temperature) at 300 K. For parallel tempering metadynamics, the system was equilibrated by 100-ps simulation in the NPT ensemble at 300 K, followed by 100-ps simulation in the NVT ensemble (constant number of particles, volume, and temperature) at each temperature used in parallel tempering metadynamics (278, 287, 295, 303, 312, 321, 329, 338, 346, 355, 365, 375, 385, 396, 406, 416, 427, 437, 448, 459, 470, 482, 493, 505, 517, 528, 539, 551, 562, 573, 584, and 595 K).

### 2.3 Metadynamics With the AlphaFold Collective Variable

Molecular dynamics simulation makes it possible to realistically model the evolution of a molecular system. Due to its high computational cost, it can sample relatively short time scales, typically nanoseconds or microseconds for explicitly solvated proteins. This is usually not enough to accurately predict the long-term distribution of states of the system studied.

Multiple methods have been introduced to address this problem. One group of methods involves artificial forces or potentials to help the system cross energy barriers (Spiwok et al., 2015). The system, instead of being stuck in a single local energy minimum, explores multiple energy minima. Metadynamics (Laio and Parrinello, 2002) achieves this by “flooding” energy minima by a history-dependent bias potential.

Another group of methods involves elevated temperatures to cross energy barriers. This is the basis of parallel tempering (Sugita and Okamoto, 1999). The system is simulated in multiple replicas at different temperatures. These replicas can occasionally swap their coordinates based on predefined criteria. As a result, sampling of states at the temperature of interest is more efficient than conventional simulation. Parallel tempering and metadynamics have been successfully combined into parallel tempering metadynamics (Bussi et al., 2006).

This bias potential in metadynamics and parallel tempering metadynamics is defined as a function of collective variables (CVs). A collective variable is a descriptor of the molecular system studied predefined by the user. It must be a differentiable function of the atomic coordinates. Furthermore, its value should reflect the state of the simulated system, including metastable states. In this work, we introduce a novel AlphaFold-based CV, and we use it as a sole CV or together with *α*-RMDS CV (Pietrucci and Laio, 2009).

**FIGURE 1:**
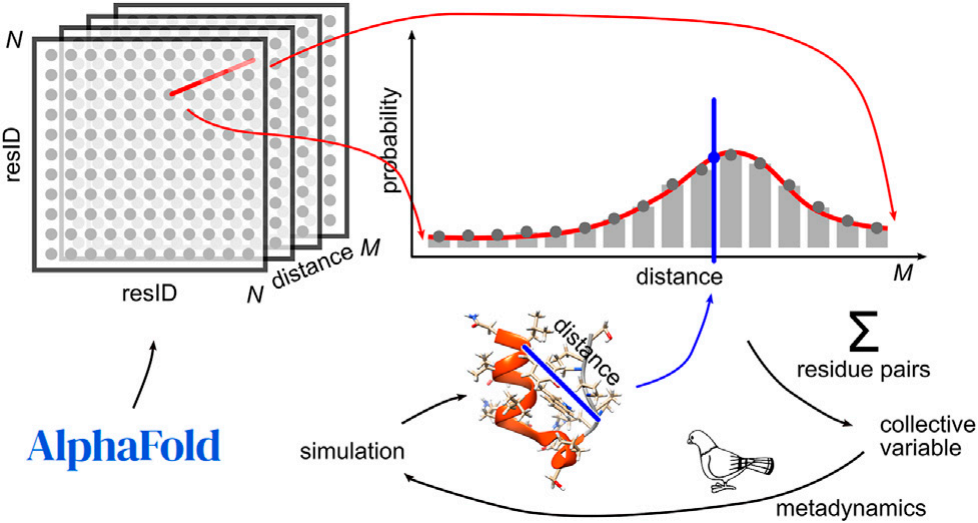
Schematic representation of AlphaFold-based CV (AlphaFold logo - credit to Deepmind).

Calculation of AlphaFold-based CV is presented in Figure 1. One of the outputs of AlphaFold 2 is a tensor *D* of distance probabilities with dimension *N* × *N* × *M*, where *N* is the number of residues and *M* is the number of distance bins (by default 64). For each residue pair, this tensor was converted from logits to probabilities *D* [*i*, *j*, *k*] (where *i*, *j* are the indexes of residues and *k* is an index of a bin) and scaled to yield the sum of probabilities along the *k* axis equal to 1.

Given a fixed conformation *C* of the molecule, we can denote ${\hat{d}}_{i,j}$ the index of the distance bin where the distance of the residues *i*, *j* reduces. Then, the random variable *R*_*i*,*j*_ with discrete values 0, 1 and the meaning “for a randomly chosen conformation, the distance between *i* and *j* falls into the bin ${\hat{d}}_{i,j}$ ” yields an expected value $ER_{i,j}=D\left[i,j,{\hat{d}}_{i,j}\right]$ . Hence, by linearity of expectation, the random variable *R* with the meaning “number of inter-residual distances of a randomly chosen conformation that fall into the same bin as with *C*” yields the expected value:

$$ER=\overset{N}{\underset{i=1}{\sum }}\overset{i-1}{\underset{j=1}{\sum }}D\left[i,j,{\hat{d}}_{i,j}\right].$$ (1)

Therefore, the sum can be interpreted as a probabilistically based measure to assess how AlphaFold would favor the conformation *C*.

In order to be used in metadynamics, the CV is desired to be smooth and must be differentiable with respect to atomic coordinates. There are many interpolation techniques to choose from, and we use an approach derived from path collective variable definitions (path CV) (Branduardi et al., 2007; Spiwok and Králová, 2011), simplified to the one-dimensional case. Assuming *d* to be an inter-residue distance between *i*, *j* in our conformation *C*, we calculate

$$P_{i,j}\left(d\right)=\frac{\sum_{k=1}^{M}D\left[i,j,k\right]e^{-\lambda {\left(d-d_{k}\right)}^{2}}}{\epsilon +\sum_{k=1}^{M}e^{-\lambda {\left(d-d_{k}\right)}^{2}}},$$ (2)

where *d*_*k*_ is the inter-residue distance of the *k*th bin in *D*. Other techniques (polynomial spline interpolation, etc.) could appear less complicated. However, when considering the required differentiation, our approach is computationally efficient (the exponential terms are reused) and less error-prone for implementation.

The value of *λ* must be determined empirically–low values make the curve smoother and high values favor *P*_*i*_s more strictly. We used *λ* = 1,000 nm^−2^ in our calculations. The constant *ϵ* (pseudocount, not used in the original path CVs) was introduced to improve numerical stability when *d* falls out of the *d*_*k*_s range and Eq. 2 approaches 0/0. Finally, the values of *P*_*i*,*j*_(*d*) were calculated for all C*α*–C*α* distances measured during the simulation and summed according to Eq. 1. The result was used as a collective variable in metadynamics simulations.

The AlphaFold output (the final model and the corresponding pickle file) was converted by a Python script provided at GitHub (https://github.com/spiwokv/af2cv). It converts the 3D structure (in Gromacs format) and the pickle file into a Plumed input by the following command:

python af2cv.py model.gro model.pkl > plumed.dat

The resulting output (plumed.dat) must be modified for the given type of calculation, for example, for monitoring of the CV, for metadynamics, or for other free-energy modeling methods available in Plumed.

In metadynamics, we used either AlphaFold-based CV or its combination with *α*-RMSD. Metadynamics floods the free-energy minima by bias potential comprising Gaussian hills. The widths of these hills were 0.1 (Trp-cage) or 0.04 (*β* hairpin) in the direction of AlphaFold-based CV and 0.1 in the direction of *α*-RMSD. Well-tempered metadynamics (Barducci et al., 2008), which reduces the heights of hills with the progress of the simulation, was used. The height of the hills was set to 0.5 kJ/mol, and the bias factor was set to 8.

Parallel tempering metadynamics (Bussi et al., 2006) also used either AlphaFold-based CV or its combination with *α*-RMSD. The widths and heights of the hills and the bias factor were the same as in metadynamics. Replica exchange attempts were made every 1 ps. Free-energy surfaces were calculated using Metadynminer (Trapl and Spiwok, 2020). 3D structures were visualized by UCSF Chimera (Pettersen et al., 2004).

## 3 Results

### 3.1 Trp-Cage

**FIGURE 2:**
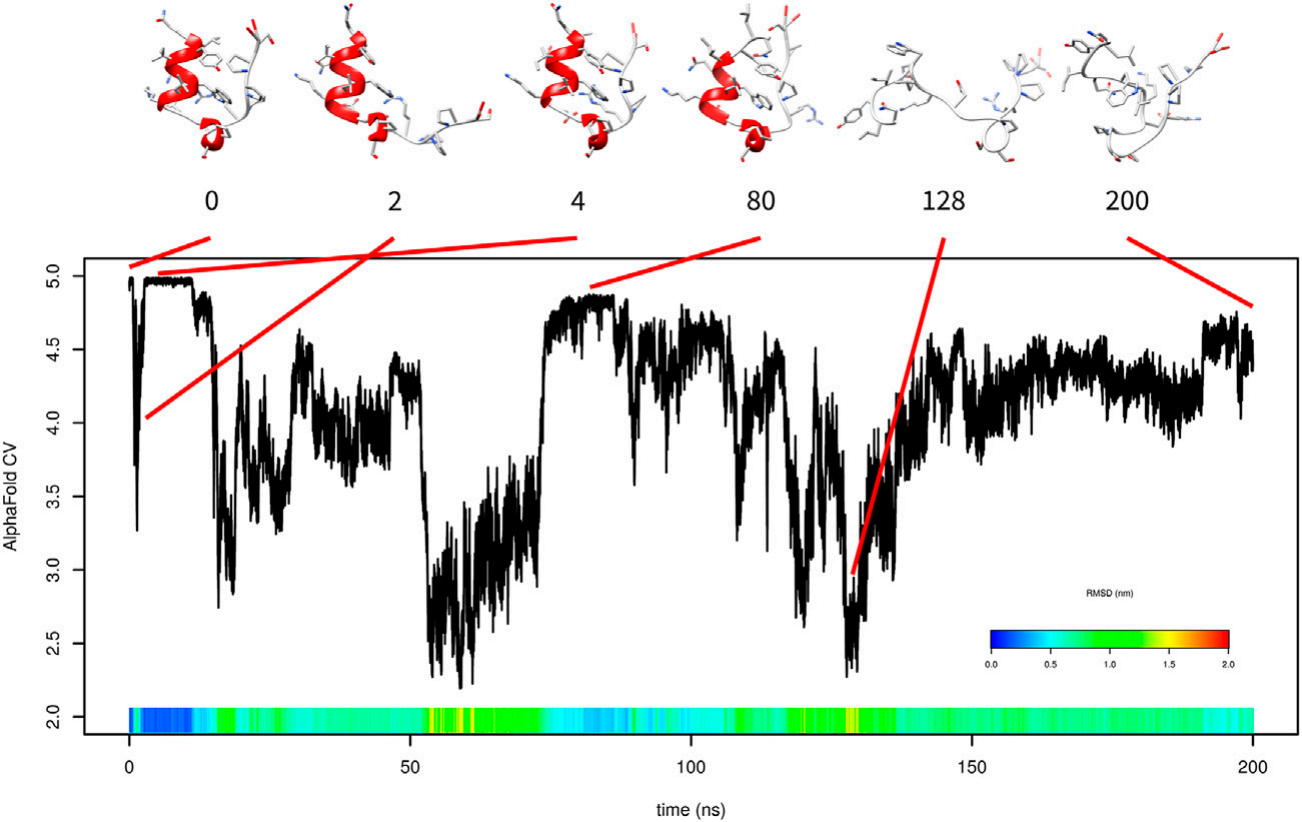
Evolution of AlphaFold-based CV in metadynamics simulation of Trp-cage with AlphaFold-based CV with selected frames. RMSD is depicted in the color scale.

First, 200-ns metadynamics was performed only with AlphaFold-based CV (Figure 2). The simulation started from the folded state. The folded state corresponds to AlphaFold-based CV values between 4.9 and 5. After approximately 600 ps, it partially unfolded and AlphaFold-based CV dropped to approximately 3. This unfolding consisted of detachment of the C-terminal tail from the N-terminal *α*-helix. The C-terminal tail returned at time 2.6 ns, and the structure returned to the folded state. It stayed there up to 11 ns. This is represented in Figure 2 by snapshots at 0, 2, and 4 ns.

At approximately 80 ns, the system returned to a near-native state with the AlphaFold-based CV between 4.80 and 4.85. It differed from the native state by unwinding of the three N-terminal residues from the *α*-helix and a slightly different position of the C-terminus. From the start to time 80 ns, some content of *α*-helix was present in the structure. After that, the protein lost any helix content and was not able to fold.

Unfortunately, the free-energy surface calculated by this metadynamics simulation was not realistic (data not shown) because the unfolded state was calculated as significantly more stable than the folded state. This was due to lack of folding events.

**FIGURE 3:**
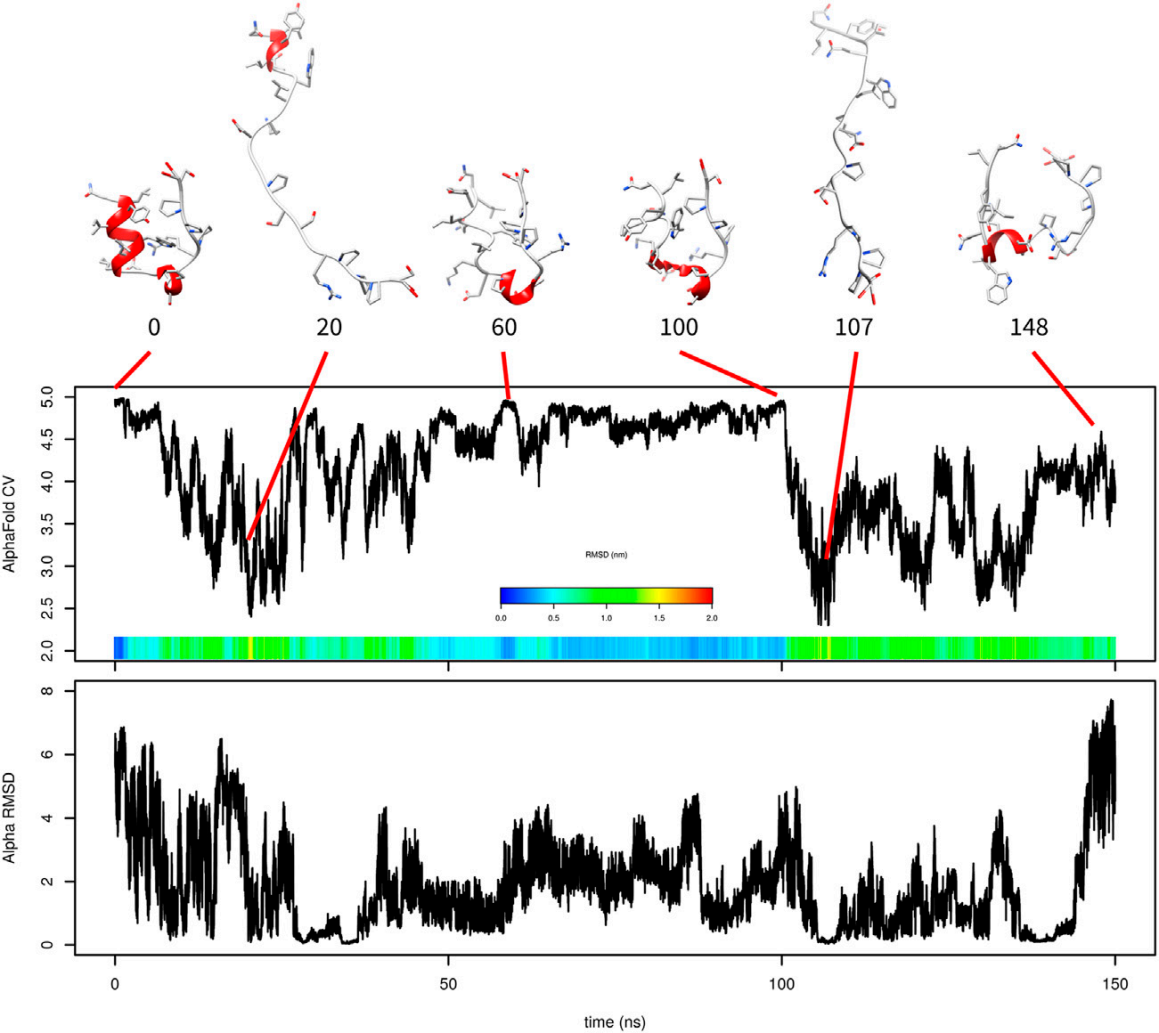
Evolution of AlphaFold-based CV and *α*-RMSD in metadynamics simulation of Trp-cage with both CVs with selected frames. RMSD is depicted in the color scale.

The simulation with one AlphaFold-based CV indicated that the formation of *α*-helix is critical and that AlphaFold-based CV itself cannot efficiently accelerate it. For this reason, we added a second CV (*α*-RMSD) to accelerate helix formation. The results of this 150 ns simulation are depicted in Figure 3.

Similar to the previous simulation, there was relatively fast unfolding. The system explored structures with high values of AlphaFold-based CV between 60 and 100 ns. The value of AlphaFold-based CV was fluctuating between 4.4 and 4.96 at this stage. These structures were very similar to those of the native state in overall shape, but the N-terminal *α*-helix was not formed.

The simulation also explored states with higher *α*-RMSD CV, which is depicted in Figure 3 at 148 ns. This structure is characterized by a helix-like structure of residues 6–13 (Figure 3 shows residues 6–9 as the helix because the remaining residues do not meet the definition of *α*-helix used by UCSF Chimera).

Again, the predicted free-energy surface was not realistic (the unfolded state was significantly more favored than the folded one; data not shown). This can be explained by the lack of folding events.

Since it was not possible to observe enough folding events to accurately predict the folding free-energy surface, we replaced metadynamics with parallel tempering metadynamics. The combination of metadynamics with parallel tempering makes it possible to accelerate degrees of freedom that are not covered by CVs.

**FIGURE 4:**
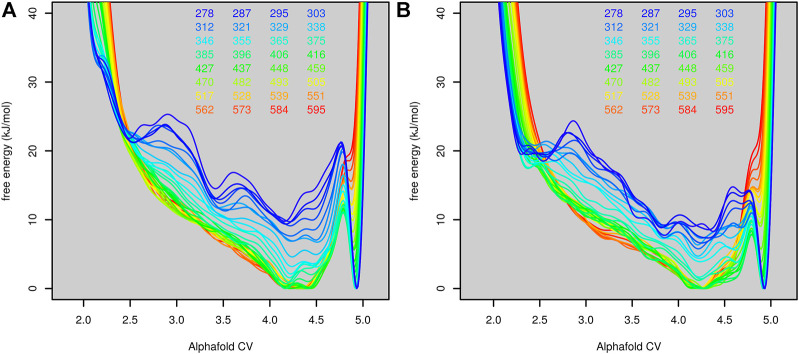
Free-energy surfaces of Trp-cage (as functions of AlphaFold-based CV) calculated at different temperatures in the run stared from the folded (**(A)**, the first run) and unfolded (**(B)**, the second run) state.

The first parallel tempering metadynamic simulation started from the native structure of the protein. Before the parallel tempering metadynamics simulation, each replica was equilibrated by 100-ps molecular dynamics simulation at the corresponding temperature. This was usually not long enough to unfold the protein, so most simulations started from the native or near-native structure. The predicted free-energy surfaces at different temperatures are depicted in Figure 4A.

The folded state is modeled as the global minimum for lower temperatures (up to 375 K, 102°C). Above this, the unfolded state was more favored. The conversion of the free-energy surface to probability (as exp(−*G*/*kT*)) and integration of the probability of AlphaFold score higher and lower than 4.75 (estimated border between the unfolded and folded state) revealed that the protein is stable in its native structure up to 329 K (56°C). This was in good agreement with the experimentally determined melting temperature (42°C).

The fact that the simulation started mostly from the folded state may bias the free-energy surface toward the folded state. To rule out this possibility, we performed another simulation (second run) starting from the first run after 10 ns. At this point, all replicas were unfolded (only replica 18 was in a state similar to the structure at 2 ns in Figure 2). All other settings were the same as in the first run. The free-energy surface is depicted in Figure 4B. Free-energy surfaces from the first and the second run are very similar. The only notable difference was in the lower barrier between the folded and unfolded states.

Free-energy surfaces calculated by parallel tempering metadynamics may be stable owing not only to transitions between different states of the system but also as an artifact of replica exchanges. Let us imagine a parallel tempering (or parallel tempering metadynamics) with just two replicas, one starting from a folded and one from an unfolded protein. High number of replica exchanges causes the single temperature trajectory switches between the folded and unfolded states, even in the absence of any real folding and unfolding events. The results of such simulations may be wrongly interpreted as an equilibrium between the folded and unfolded state.

**FIGURE 5:**
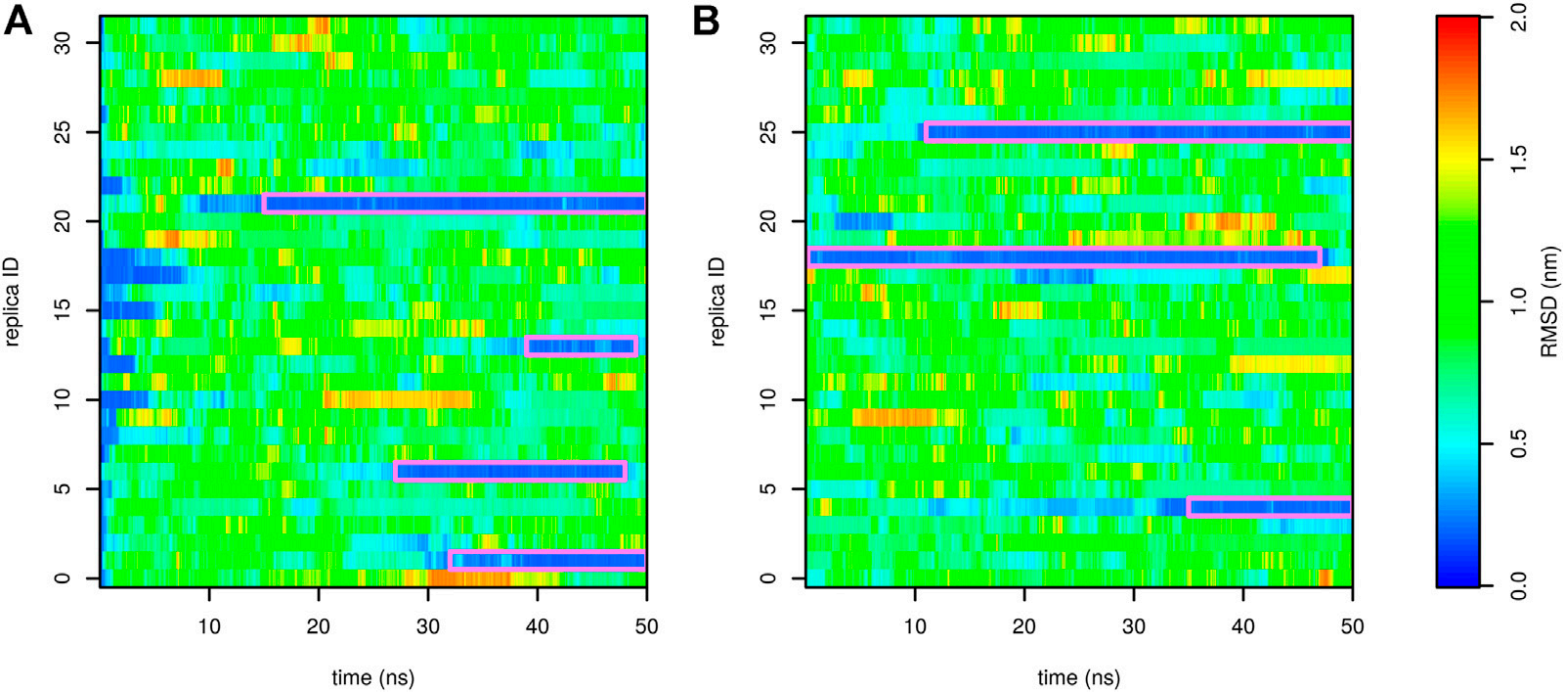
Profiles of RMSD of Trp-cage as a function of time calculated for demultiplexed trajectories from the run stared from the folded (**(A)**, the first run) and unfolded (**(B)**, the second run) state. The folded state is highlighted by a purple frame.

To avoid this artifact, we performed demultiplexing (“demuxing”) of replicas to obtain continuous trajectories, regardless of the evolution of temperature. The evolution of root-mean-square deviation (RMSD) from the native structure in demuxed trajectories is depicted in Figure 5. In the first and second runs, we observed four and three folding events, respectively. In general, there were typically two replicas in the folded state. This indicates that the folding free-energy surfaces in Figure 4 are stable and not affected by either the starting state or the number of folding events.

**FIGURE 6:**
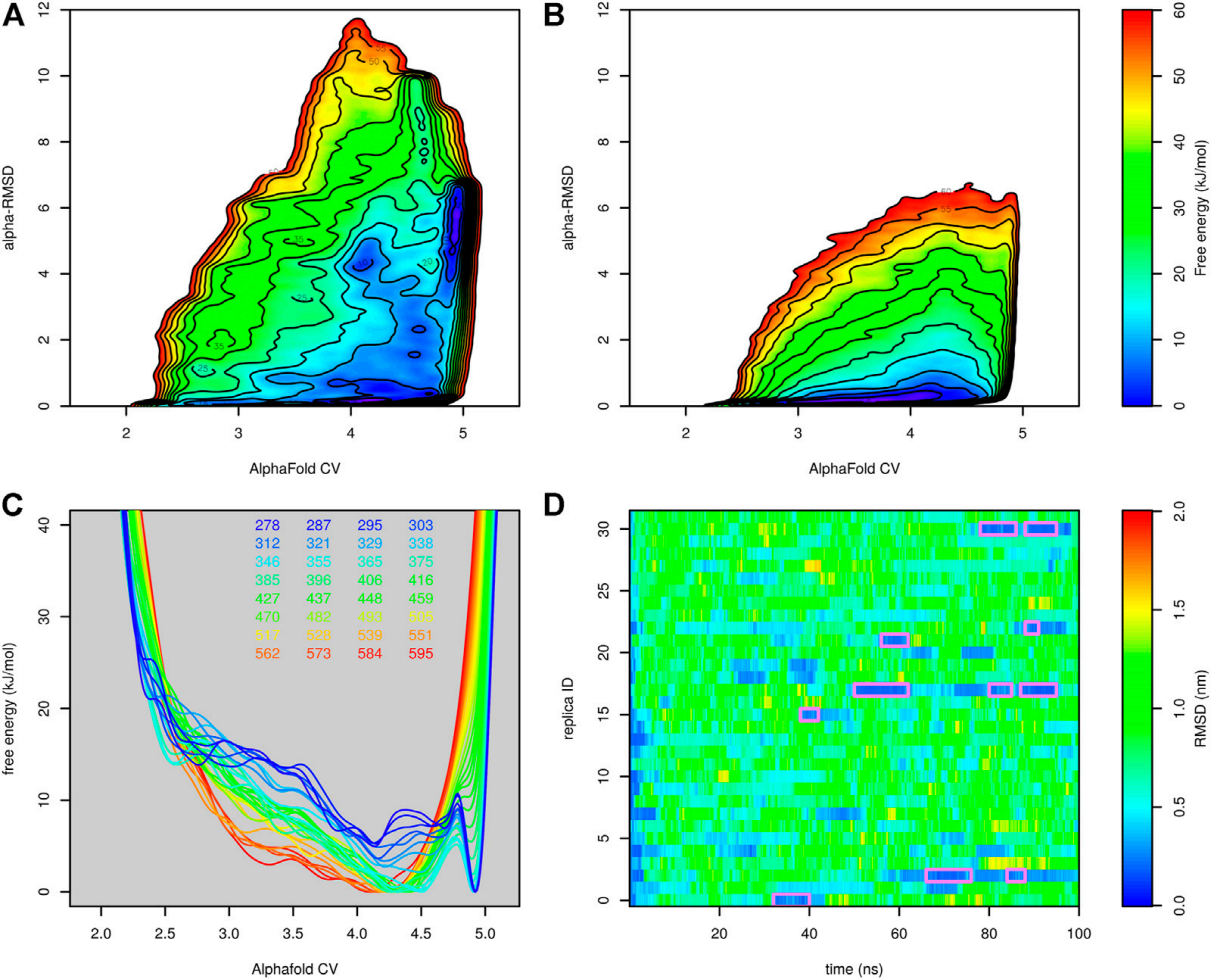
Results of parallel tempering metadynamics simulation of Trp-cage with AlphaFold-based CV and *α*-RMSD collective variables. **(A)** Free-energy surface at 303 K, **(B)** free-energy surface at 595 K, **(C)** free-energy surfaces calculated at different temperatures and **(D)** profiles of RMSD as a function of time calculated for demultiplexed trajectories.

Finally, we tested parallel tempering metadynamics with two collective variables, AlphaFold-based CV and *α*-RMSD. It was necessary to prolong the simulation from 50 to 100 ns. The results are depicted in Figure 6. Figures 6A,B compare the free-energy surface at 303 and 595 K. The former is characterized by two main minima. The minimum corresponding to the folded structure is located at AlphaFold-based CV ∼ 5 and *α*-RMSD ∼ 6. The unfolded minimum is at the bottom of the plot. There are several other notable local minima, namely, at high values of *α*-RMSD, which corresponds to a structure with a long helix, or at AlphaFold-based CV ∼ 4 and *α*-RMSD ∼ 4, which corresponds to the structure with the N-terminal helix formed but the C-terminal tail detached from the helix. At 595 K, the unfolded minimum was the only minimum of the system. Figures 6C,D are in good agreement with the results of parallel tempering simulations with AlphaFold-based CV as the only collective variable. A reasonable number of folding events were observed.

**FIGURE 7:**
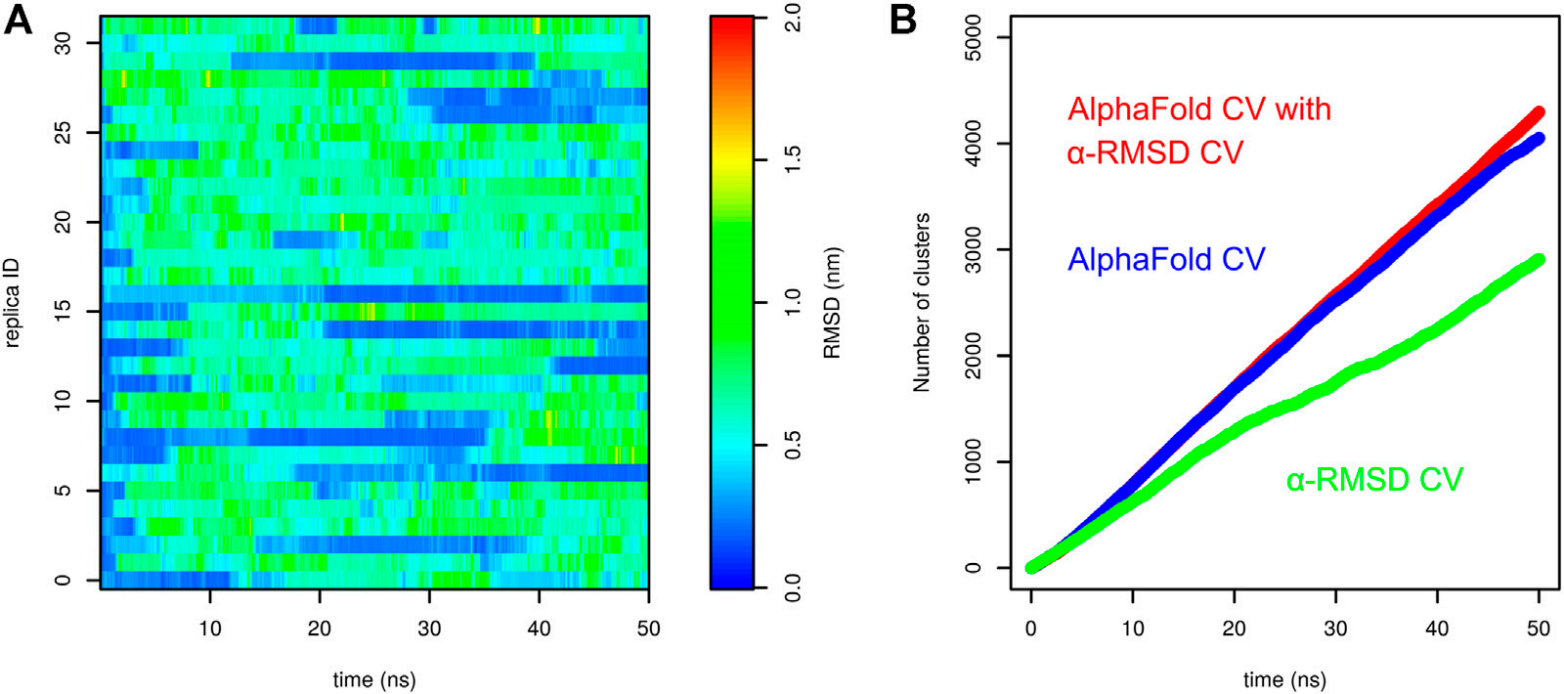
Comparison of parallel tempering metadynamics simulation of Trp-cage with *α*-RMSD CV with other CVs. **(A)** Profiles of RMSD in parallel tempering metadynamics with *α*-RMSD CV as a function of time calculated for demultiplexed trajectories and **(B)** comparison of the cumulative number of conformational clusters explored by parallel tempering metadynamics with different CVs.

In order to compare the performance of AlphaFold-based CV and its combination with *α*-RMSD, we carried out a parallel tempering simulation with *α*-RMSD as the only CV. Other parameters were the same as in the simulations with the AlphaFold-based CV. Surprisingly, we observed multiple folding events (Figure 7A). However, it is apparent that unfolded structures in these simulations do not significantly divert from the native structure. In other words, the sampling of various conformation states is much more intensive in the simulation with AlphaFold-based CV in combination of AlphaFold and *α*-RMSD CVs, compared to *α*-RMSD CVs.

This was further demonstrated by assessment of the number of conformational clusters explored in the simulation. The structures sampled in each parallel tempering simulation at 303 K were analyzed by the clustering method by Daura et al. (1999) (cutoff set to 0.1 nm). Figure 7B shows the evolution of the cumulative number of clusters. Clearly, the number of clusters sampled by *α*-RMSD CV is significantly lower than for AlphaFold-based CV. The combination of AlphaFold and *α*-RMSD CVs gives little extra sampling.

### 3.2 *β* Hairpin

**FIGURE 8:**
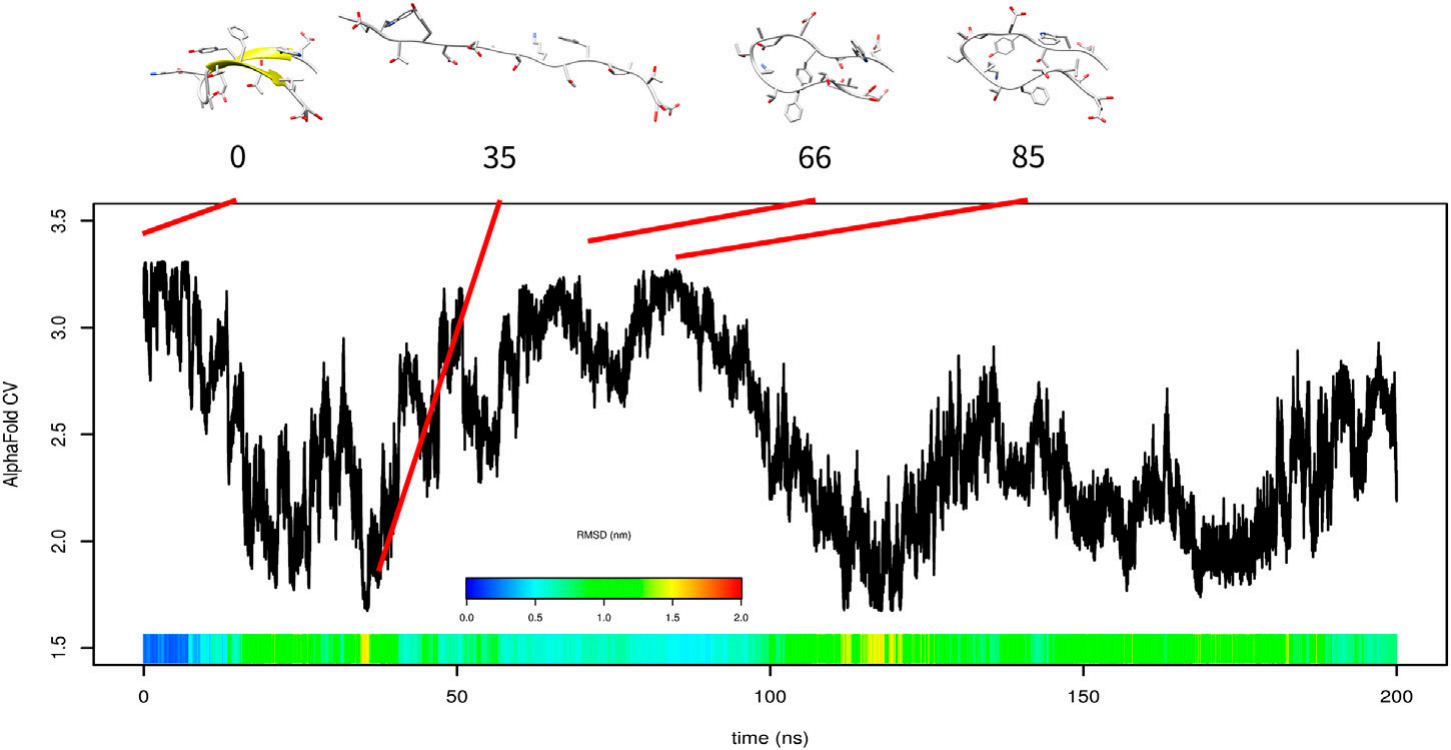
Results of metadynamics simulation of *β*-hairpin with the AlphaFold collective variable with selected structures. RMSD is depicted in the color scale.

Formation of *β*-sheet is in general slower than the formation of *α*-helix Zimm et al. (1959). Furthermore, it is more difficult to accelerate it. To evaluate the performance of AlphaFold-based CV on the formation of *β*-sheet structures, we studied folding of a model *β* hairpin mini-protein, which comprises a single antiparallel *β*-sheet. Similar to Trp-cage metadynamics simulation, after complete unfolding at the beginning of 200-ns metadynamics simulation, the system explored structures with high AlphaFold-based CV (at time 66 or 85 ns, Figure 8). These structures were spatially similar to the native structure; however, the secondary structure was formed incorrectly.

**FIGURE 9:**
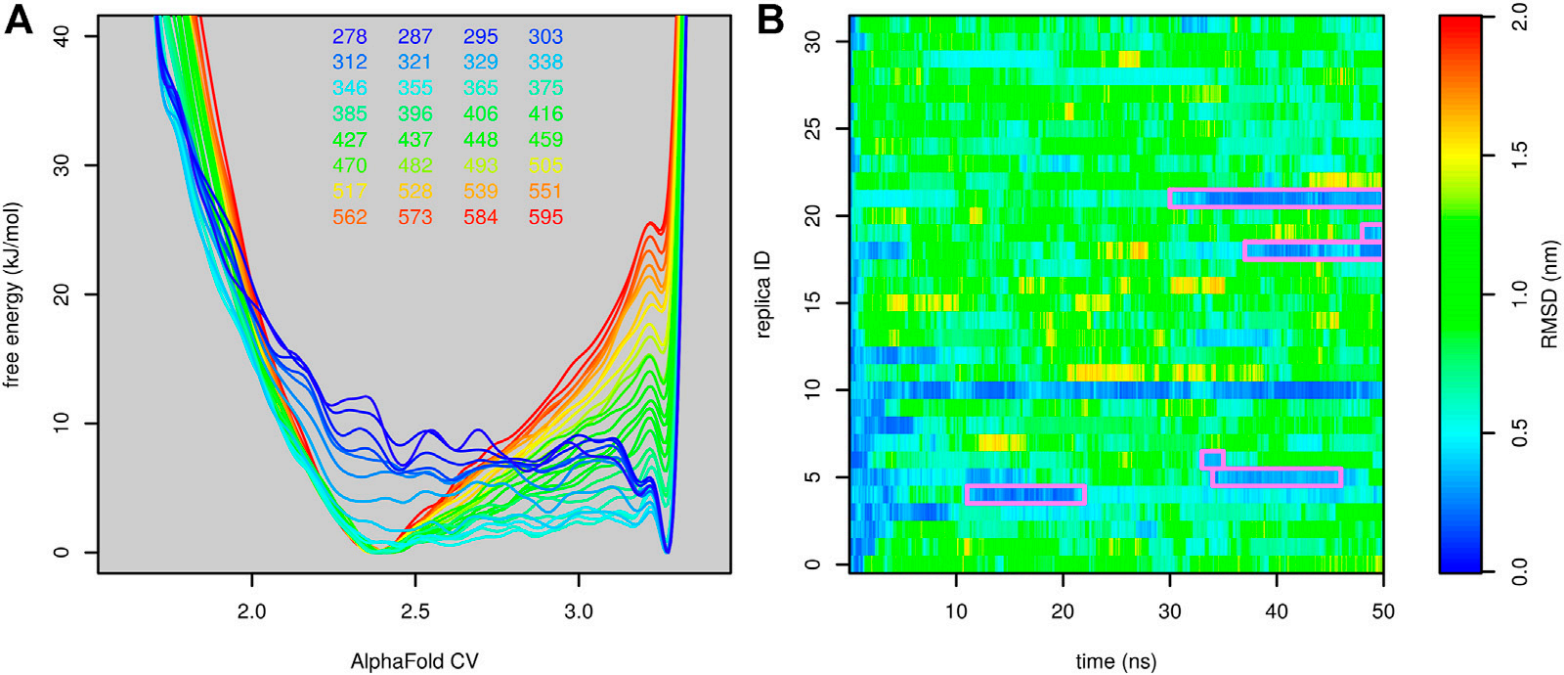
Results of parallel tempering metadynamics simulation of *β*-hairpin with AlphaFold collective variables. **(A)** Free-energy surfaces calculated at different temperatures and **(B)** profiles of RMSD as a function of time calculated for demultiplexed trajectories.

Similarly to Trp-cage, the combination of parallel tempering with metadynamics made it possible to accurately predict its free-energy surface at different temperatures and observe multiple folding events (Figure 9). *β* Hairpin was predicted to prefer the folded state at low temperatures and the unfolded state at higher temperatures. The melting temperature was estimated to be 287 K. This is in good agreement with the experimentally determined fraction of the native structure at 278 K, which is 40% Blanco et al. (1994).

## 4 Discussion

The performance of metadynamics driven by AlphaFold-based CV can be assessed by comparison with unbiased simulations, parallel tempering simulations, and metadynamics using other CVs. One of the model mini-proteins used in this study–Trp-cage–folds with the mean folding time equal to 14 *μ*s in a simulation with a similar setup (Lindorff-Larsen et al., 2011). In a parallel tempering simulation (without metadynamics) with a similar setup and the same duration (200 ns) as in our previous study, we did not observe any folding events (Trapl et al., 2019). With the neural network–approximated solvent-accessible surface area as a CV, we observed more folding events than in this study (eight compared to four or three in this study). Both studies used 200-ns parallel tempering metadynamics. However, for approximation of the solvent-accessible surface area by a neural network, it is necessary to have a series of folded and unfolded structures of the system. We obtained these from the 208-*μ*s trajectory kindly provided by D.E. Shaw Research. In contrast, AlphaFold-based CV can be built just using the sequence of a protein. Therefore, it does not suffer from the “chicken and egg” problem of the necessity of using folding trajectories to simulate folding.

Our results have shown that the critical process in the folding of Trp-cage accelerated by AlphaFold-based CV is the formation of the secondary structure. Without this, metadynamics can force formation of structures similar to the native one, but it is wrong in terms of the secondary structure. This cannot be easily solved by the *α*-RMSD CV. Replacement of metadynamics by parallel tempering metadynamics helped solve this problem. *α*-RMSD CV itself makes it possible to fold and unfold Trp-cage in a reasonable time scale; however, a significantly lower number of conformations is sampled.

AlphaFold-based CV was also tested on *β* hairpin, which was used as a representative of *β*-sheet mini-protein. Similar to the Trp-cage, AlphaFold-based CV could not fold the mini-protein in 200-ns metadynamics but was able to fold it and predict the temperature-dependent free-energy surface by parallel tempering metadynamics.

We used the concept of path CVs (Equation 2) (Branduardi et al., 2007) to convert the discrete distance probability profile into a continuous one. The path CV includes a prefactor *λ* that must be set prior to the application of the CV. Here, we set *λ* equal to 1,000 nm −2. This value was chosen based on the plot of the sample probability profiles (data not shown). We believe that the same value of *λ* can be used in studies of other proteins because the distance values, for which these profiles are constructed, are the same or very similar (the Supplementary Information of the reference (Jumper et al., 2021) states “The bins cover the range from 2 Å to 22 Å”).

The parameter *ϵ* used in Eq. 2 has not been used, to our knowledge, in the context of path CV. It can be used to make the simulation more stable by elimination of a high gradient when the numerator and the denominator of Eq. 2 are close to zero. In this study, we used this equation to approximate a probability. In general applications of path CVs, this coefficient may cause artifacts and must be used carefully.

In parallel tempering and parallel tempering metadynamics, it is necessary to keep the size of a simulation box small. This is because the potential energy distribution in large systems (large boxes with large numbers of water molecules) is relatively narrow as a result of an averaging effect. This causes the overlap of potential energy histograms of two neighboring replicas to be small, which causes a low probability of coordinate exchange and thus poor performance of parallel tempering. On the other hand, small box size increases the risk of artifacts caused by interactions of the simulated protein with its replicas from the neighboring periodic boxes. The trajectories from parallel tempering metadynamics simulations were visually inspected (data not shown). This revealed that self-interactions are relatively rare and are limited to head-to-tail interactions of fully unfolded proteins. Therefore, we believe that self-interactions do not cause any significant artifacts.

A similar approach as presented here was applied by Nassar et al. (2022). Using the output from other machine learning–based protein structure modeling tools, they folded multiple significantly larger proteins, however, in substantially longer simulations.

In principle, it is possible to use AlphaFold to predict the native structure of the studied protein and then use RMSD from the predicted native structure as a collective variable (here *R* ^2^ of RMSD from the native structure and the AlphaFold CV is 0.83 for Trp-cage). We see two major differences between the RMSD from the native structure and AlphaFold CV. First, bell-shaped distance probability profiles have different widths for different residue pairs. Narrow profiles give rise to higher energy gradients; thus, they have higher priority than wide profiles. We believe that this prioritization of residue pairs may play a role in CV performance.

Second, AlphaFold-based CV is an example of a “soft” collective variable. It is to be recalled that the AlphaFold-based CV represents the expected number of pairs of residues whose distance matches that of the current structure. Thus, it seems very likely that the local minima in a fixed basis of attraction could have similar AlphaFold-based CV values. If true, the AlphaFold-based CV would change only if the simulation left the initial basis of attraction. On the contrary, the RMSD of the native structure is a variable that increases continuously with divergence from the native structure. Hence, it can vary much even when restricted to minima on a fixed basis of attraction. This could cause AlphaFold-based CV to provide faster divergence from the initial structure.

A similar behavior could be expected from other collective variables that do not utilize the native structure, such as *α*-RMSD. As our experimental comparison shows, *α*-RMSD was capable of guiding the simulations into several folding events; however, it could not lead it into a very divergent basis of attraction. In contrast, the AlphaFold-based CV seems to have similar values on similar bases of attraction, causing the simulation to move faster toward very different configurations. The very same property, of course, prevents it from convergence to minima in different bases of attraction; hence, a second CV is needed to ensure the convergence.

In the future, we can imagine a more focused version of AlphaFold-based CV. Since it is not the purpose of an AlphaFold CV–driven simulation to predict the structure of a protein, which can be carried out much more efficiently by AlphaFold itself, we see its application in refinement focused on protein loops, domains, and pockets, for example, to accelerate induced fit in docking simulations. Instead of calculating the sum of *P*(*d*) across all residues, the sum can be calculated on a predefined set of residues. This would make it possible to focus AlphaFold-based CV to a certain part of the protein. Analogously, it would be possible to split the AlphaFold-based CV into two CVs, one focused locally (that is, on the secondary structure) and one globally.

## Data Availability

The datasets presented in this study can be found in online repositories. The names of the repository/repositories and accession number(s) can be found at: https://doi.org/10.5281/zenodo.6122678.

## References

[B1] Abraham M. J. Murtola T. Schulz R. Páll S. Smith J. C. Hess B. (2015). GROMACS: High Performance Molecular Simulations through Multi-Level Parallelism from Laptops to Supercomputers. SoftwareX 135, 224504. 10.1016/j.softx.2015.06.001

[B2] Barducci A. Bussi G. Parrinello M. (2008). Well-tempered Metadynamics: A Smoothly Converging and Tunable Free-Energy Method. Phys. Rev. Lett. 100, 020603. 10.1103/PhysRevLett.100.020603 18232845

[B3] Bernstein F. C. Koetzle T. F. Williams G. J. B. Meyer E. F. Brice M. D. Rodgers J. R. (1977). The Protein Data Bank: a Computer-Based Archival File for Macromolecular Structures. J. Mol. Biol. 112, 535–542. 10.1016/S0022-2836(77)80200-3 875032

[B4] Blanco F. J. Rivas G. Serrano L. (1994). A Short Linear Peptide that Folds into a Native Stable β-hairpin in Aqueous Solution. Nat. Struct. Mol. Biol. 1, 584–590. 10.1038/nsb0994-584 7634098

[B5] Branduardi D. Gervasio F. L. Parrinello M. (2007). From *A* to *B* in Free Energy Space. J. Chem. Phys. 126, 054103. 10.1063/1.2432340 17302470

[B6] Bussi G. Donadio D. Parrinello M. (2007). Canonical Sampling through Velocity Rescaling. J. Chem. Phys. 126, 014101. 10.1063/1.2408420 17212484

[B7] Bussi G. Gervasio F. L. Laio A. Parrinello M. (2006). Free-Energy Landscape for β Hairpin Folding from Combined Parallel Tempering and Metadynamics. J. Am. Chem. Soc. 128, 13435–13441. 10.1021/ja062463w 17031956

[B8] Darden T. York D. Pedersen L. (1993). Particle Mesh Ewald: An N.Log(N) Method for Ewald Sums in Large Systems. J. Chem. Phys. 98, 10089–10092. 10.1063/1.464397

[B9] Daura X. Gademann K. Jaun B. Seebach D. van Gunsteren W. F. Mark A. E. (1999). Peptide Folding: When Simulation Meets Experiment. Angew. Chem. Int. Ed. 38, 236–240. 10.1002/(sici)1521-3773(19990115)38:1/2<236::aid-anie236>3.0.co;2-m

[B10] Gronenborn A. M. Filpula D. R. Essig N. Z. Achari A. Whitlow M. Wingfield P. T. (1991). A Novel, Highly Stable Fold of the Immunoglobulin Binding Domain of Streptococcal Protein G. Science 253, 657–661. 10.1126/science.1871600 1871600

[B11] Hess B. Bekker H. Berendsen H. J. C. Fraaije J. G. E. M. (1997). LINCS: A Linear Constraint Solver for Molecular Simulations. J. Comput. Chem. 18, 1463–1472. 10.1002/(sici)1096-987x(199709)18:12<1463::aid-jcc4>3.0.co;2-h

[B12] Jorgensen W. L. Chandrasekhar J. Madura J. D. Impey R. W. Klein M. L. (1973). Comparison of Simple Potential Functions for Simulating Liquid Water. J. Chem. Phys. 79, 926–935. 10.1063/1.445869

[B13] Jumper J. Evans R. Pritzel A. Green T. Figurnov M. Ronneberger O. (2021). Highly Accurate Protein Structure Prediction with AlphaFold. Nature 596, 583–589. 10.1038/s41586-021-03819-2 34265844 PMC8371605

[B14] Laio A. Parrinello M. (2002). Escaping Free-Energy Minima. Proc. Natl. Acad. Sci. U.S.A. 99, 12562–12566. 10.1073/pnas.202427399 12271136 PMC130499

[B15] Lindorff-Larsen K. Piana S. Dror R. O. Shaw D. E. (2011). How Fast-Folding Proteins Fold. Science 334, 517–520. 10.1126/science.1208351 22034434

[B16] Lindorff-Larsen K. Piana S. Palmo K. Maragakis P. Klepeis J. L. Dror R. O. (2010). Improved Side-Chain Torsion Potentials for the Amber ff99SB Protein Force Field. Proteins 78, 1950–1958. 10.1002/prot.22711 20408171 PMC2970904

[B17] Nassar R. Brini E. Parui S. Liu C. Dignon G. L. Dill K. A. (2022). Accelerating Protein Folding Molecular Dynamics Using Inter-residue Distances from Machine Learning Servers. J. Chem. Theory Comput. 18, 1929–1935. 10.1021/acs.jctc.1c00916 35133832 PMC9281603

[B18] Neidigh J. W. Fesinmeyer R. M. Andersen N. H. (2002). Designing a 20-residue Protein. Nat. Struct. Biol. 9, 425–430. 10.1038/nsb798 11979279

[B19] Parrinello M. Rahman A. (1981). Polymorphic Transitions in Single Crystals: A New Molecular Dynamics Method. J. Appl. Phys. 52, 7182–7190. 10.1063/1.328693

[B20] Pettersen E. F. Goddard T. D. Huang C. C. Couch G. S. Greenblatt D. M. Meng E. C. (2004). UCSF Chimera—A Visualization System for Exploratory Research and Analysis. J. Comput. Chem. 25, 1605–1612. 10.1002/jcc.20084 15264254

[B21] Pietrucci F. Laio A. (2009). A Collective Variable for the Efficient Exploration of Protein Beta-Sheet Structures: Application to SH3 and GB1. J. Chem. Theory Comput. 5, 2197–2201. 10.1021/ct900202f 26616604

[B22] Sanchez-Pulido L. Ponting C. P. (2021). Extending the Horizon of Homology Detection with Coevolution-Based Structure Prediction. J. Mol. Biol. 433, 167106. From Protein Sequence to Structure at Warp Speed: How Alphafold Impacts Biology. 10.1016/j.jmb.2021.167106 34139218 PMC8527833

[B23] Senior A. W. Evans R. Jumper J. Kirkpatrick J. Sifre L. Green T. (2020). Improved Protein Structure Prediction Using Potentials from Deep Learning. Nature 577, 706–710. 10.1038/s41586-019-1923-7 31942072

[B24] Spiwok V. Králová B. (2011). Metadynamics in the Conformational Space Nonlinearly Dimensionally Reduced by Isomap. J. Chem. Phys. 135, 224504. 10.1063/1.3660208 22168700

[B25] Spiwok V. Sucur Z. Hosek P. (2015). Enhanced Sampling Techniques in Biomolecular Simulations. Biotechnol. Adv. 33, 1130–1140. 10.1016/j.biotechadv.2014.11.011 25482668

[B26] Sugita Y. Okamoto Y. (1999). Replica-exchange Molecular Dynamics Method for Protein Folding. Chem. Phys. Lett. 314, 141–151. 10.1016/S0009-2614(99)01123-9

[B27] The PLUMED consortium (2019). Promoting Transparency and Reproducibility in Enhanced Molecular Simulations. Nat. Methods 16, 670–673. 10.1038/s41592-019-0506-8 31363226

[B28] Trapl D. Horvacanin I. Mareska V. Ozcelik F. Unal G. Spiwok V. (2019). Anncolvar: Approximation of Complex Collective Variables by Artificial Neural Networks for Analysis and Biasing of Molecular Simulations. Front. Mol. Biosci. 6, 25. 10.3389/fmolb.2019.00025 31058167 PMC6482212

[B29] Trapl D. Spiwok V. (2020). Analysis of the Results of Metadynamics Simulations by Metadynminer and Metadynminer3d. [Dataset]. 10.48550/arXiv.2009.02241

[B30] Tribello G. A. Bonomi M. Branduardi D. Camilloni C. Bussi G. (2014). PLUMED 2: New Feathers for an Old Bird. Comput. Phys. Commun. 185, 604–613. 10.1016/j.cpc.2013.09.018

[B31] Zimm B. H. Doty P. Iso K. (1959). Determination of the Parameters for Helix Formation in Poly-*γ*-Benzyl-L-Glutamate. Proc. Natl. Acad. Sci. U.S.A. 45, 1601–1607. 10.1073/pnas.45.11.1601 16590552 PMC222768

